# Influences of the Differences Between Mothers’ and Children’s Perceptions of Parenting Styles

**DOI:** 10.3389/fpsyg.2020.552585

**Published:** 2020-10-27

**Authors:** Jiwon Cho, Jung Hee Ha, Juliet Jue

**Affiliations:** ^1^Graduate School of Counseling Psychology, Hanyang University, Seoul, South Korea; ^2^Department of Art Therapy, Hanyang Cyber University, Seoul, South Korea

**Keywords:** parenting style, perceptual differences, depression, aggression, ego-resilience

## Abstract

In this study, we explored the differences between mothers’ and children’s perceptions of mothers’ parenting styles (DMCP of MPS) and examined the effects of these differences on children’s depression, aggression, and ego-resilience. A total of 233 pairs of mothers and teen-aged children participated in the study. Our analysis produced four main findings. First, the mothers perceived their parenting attitudes as more rational and affectionate than their children did; children whose mothers rated their parenting styles more favorably had higher levels of depression and aggression and lower ego-resilience. Second, the correlation analysis and the structural equation model verification confirmed that as the DMCP of MPS increased, children’s levels of depression and aggression increased, and their ego-resilience decreased. Third, ego-resilience partly mediated the relationship between DMCP-Rationality and depression. Lastly, we found that ego-resilience and depression had dual mediation effects on the relationship between DMCP-Rationality and children’s aggression. This paper concludes with a discussion of the implications of these findings and suggestions for future studies.

## Introduction

Adolescents’ families significantly impact their psychological development. Main caregivers’ parenting styles and the quality of interactions within families have the greatest influence on children’s personality formation and interpersonal relationships (Ku, 2013; Park et al., 2014). Parenting style is defined as the attitude of parents when they respond to their children and the emotional atmosphere of their disciplinary interactions (Olivari et al., 2015). Parenting styles can affect adolescents’ emotional and behavioral problems; in particular, negative parenting styles can aggravate interpersonal relationships and cause depression, lower self-esteem, and even lower achievement among adolescents (Dwairy, 2004).

Schaefer (1959) defined parenting style based on two basic dimensions: love–hostility and autonomy–control. Meanwhile, Oh and Lee (1982) identified four dimensions of parenting style: affection (affection vs. hostility), autonomy (autonomy vs. control), achievement (achievement vs. non-achievement), and rationality (rationality vs. irrationality). Affectionate parents are receptive and devoted; they praise and encourage their children. Autonomous parents respect their children’s opinions and encourage them to think and solve problems independently rather than over-controlling them. Achievement-driven parents set high goals, and encourage and support their children’s pursuit of these goals. Rational parents value the reasoning and motivations of their children’s behaviors and thoughts.

The importance and the influence of parenting style make evaluating and understanding it crucial. However, results may differ depending on the person evaluating the parenting style. In other words, parents may self-assess their own parenting styles differently than their children. Generally, parents tend to evaluate their parenting styles more positively than their children (Kwon, 2009; Yoo, 2018). In particular, research has shown that, in terms of rationality, mothers evaluate their parenting styles as more reasonable than their children do. In addition, research has shown that mothers’ evaluations of their parenting styles in the autonomous and achievement dimensions are more positive than their children’s evaluations (Kwon, 2009).

Most studies of parenting styles have focused on children’s assessments because children’s experiences of their parents’ parenting styles differ from their parents’ expectations or intentions (Kim and Baik, 2000; Oh and Kong, 2007; Hale et al., 2008; Park and Yoo, 2011; Ku, 2013; Park et al., 2014; Hong, 2018; Jeong, 2018). Recently, researchers have given more attention to the perceptual differences between parents and children. Differences in evaluations of parenting styles between parents and children can serve as implicit clues in estimating parent–child conflicts and children’s psychological problems. The greater the differences in evaluations of parenting styles between parents and children, the more psychological and adjustment problems children tend to have. Research has shown that greater perceptual differences increase children’s emotional maladjustment and lower their self-esteem (Park et al., 2002); increase their interpersonal problems, their internalization and externalization of problems, and their adaptation difficulties (Nam and You, 2007; Yoo, 2018); and lower their self-efficacy, increase their emotional control problems, and decrease their communication capacities (Chyung, 2002; Youn, 2014; Ryu, 2016). Another study found that adolescents who rated their mothers’ parenting styles more positively than their mothers had fewer behavioral problems and higher levels of self-confidence; that study also showed that these adolescents communicated more smoothly with their mothers than adolescents who rated their mothers’ parenting styles lower than their mothers’ evaluations (Chyung, 2002).

Based on these previous studies, we examined how the differences between mothers’ and children’s perceptions of mothers’ parenting styles (DMCP of MPS) affect children’s depression and aggression.

In addition to examining adolescent depression and aggression, we decided to investigate ego-resilience. Ego-resilience refers to the capacity to respond flexibly when faced with situations that cause frustration and stress in relationships (Park et al., 2014); it plays a key role in adolescents’ emotional and behavioral issues (Cho and Lee, 2007). A previous study found that middle school students’ perceptions of their parents’ parenting styles can affect their ego-resilience (Lee and Shin, 2006). Adolescents with high ego-resilience have been shown to have fewer psychopathological problems such as depression, anxiety, anger expression, and aggression, and to have sound interpersonal relationships and school adaptation (Pulkkinen, 1996; Hart et al., 1997; Asendorpf and van Aken, 1999; Park and Lee, 2010; Song and Hwang, 2017). The higher adolescents’ ego-resilience are, the less they will experience social withdrawal and depression (Fredrickson et al., 2003; Kim et al., 2017) and the less they will display aggression (Kim and Kang, 2020). Additionally, depression in adolescents is closely related to aggression (Hale et al., 2008; Jue and Ha, 2019; Lee et al., 2020).

**FIGURE 1:**
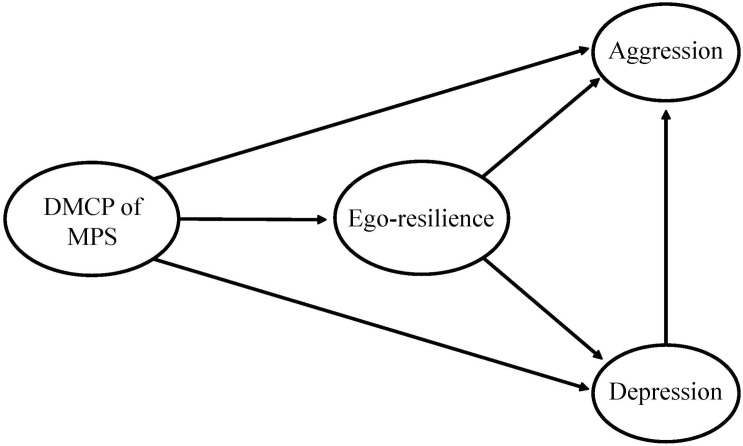
The research model.

Figure 1 presents the research model we developed; our research questions were as follows. First, what are the differences between mothers’ and children’s perceptions of mothers’ parenting styles? Second, what is the relationship between DMCP of MPS, children’s depression, aggression, and ego-resilience? Third, does children’s resilience have a mediating effect on the relationship between DMCP of MPS and children’s (a) depression and (b) aggression? Lastly, do children’s resilience and depression have a sequential dual mediating effect on the relationship between DMCP of MPS and aggression?

## Materials and Methods

### Participants

The Korean educational system consists of 6 years of elementary school, 3 years of middle school, 3 years of high school, and 4 years of university. A sample of middle school students and their mothers participated in this study. The adolescent sample consisted of 114 male (48.9%) and 119 female (51.1%) participants. Their ages ranged from 13 to 15 years old. Their areas of residence included metropolitan areas (90.1%) and local provinces (9.9%).

### Procedures

We distributed the questionnaire online and in through on-the-spot surveys. For the on-the-spot surveys, we contacted several middle schools and requested their cooperation in our research. We visited the schools that agreed to take part and explained the purpose, methods, and procedures of the study to the school teachers. Then, we visited each class, explained the study, and assured participants of their rights, including spontaneity and anonymity. Students who agreed to participate in the study completed the student questionnaires and delivered the mother questionnaires to their mothers. The questionnaires were numbered in mother–children pairs and did not require any other personally identifiable information. Both the mother and child participants signed consent forms. For the online survey, we recruited participants from several internet communities, and employed all the same procedures as in the on-the-spot surveys.

We collected responses from 243 adolescents and 282 mothers. We excluded the questionnaires that were not completed in pairs or were incomplete. Ultimately, we used a total of 233 pairs of surveys for the analysis.

### Measures

#### The Mother’s Parenting Style Scale

Oh and Lee (1982) developed the Mother’s Parenting Style Scale (MPSS) based on Schaefer’s (1959) parenting model. Originally, the scale included 50 items; Lim (1988) revised it into a 40-item scale by deleting repetitive items. The scale has four sub-scales that each consist of 10 items: *affection–hostility*, *rationality–irrationality*, *autonomy–control*, and *achievement–non-achievement.*

The questionnaires for mothers and children had the same content. Children selected the answers that best reflected their perceptions of their mothers’ parenting attitudes, and mothers selected the answers that best reflected their own perceptions of their parenting attitudes. Participants rated responses on five-point Likert scales (1 = *strongly disagree*, 5 = *strongly agree*). Higher scores meant stronger affection, rationality, autonomy, and achievement, while the lower scores meant stronger hostility, irrationality, control, and non-achievement.

The Cronbach’s αs in Lim’s study (1988) were as follows: *affection–hostility* 0.78, *rationality–irrationality* 0.81, *autonomy–control* 0.72, and *achievement–non-achievement* 0.72. In this study, we found similar levels of reliability: *affection–hostility* 0.80, *rationality–irrationality* 0.82, *autonomy–control* 0.74, and *achievement–non-achievement* 0.67.

We calculated DMCP of MPS by subtracting children’s MPSS scores from their mothers’ MPSS scores.

#### The Ego-Resiliency Scale

We employed the Ego-Resiliency Scale developed by Block and Kremen (1996) and translated into Korean and revised by Yoo and Shim (2002). The scale has a total of 14 items, evaluating adaptation to new situations, overcoming crises, and optimistic attitudes. Participants rated the items on four-point Likert scales. The higher the sum score, the greater the ego-resiliency. Block and Kremen (1996) found a Cronbach’s α of 0.76 for this scale, and Yoo and Shim (2002) reported it to be 0.67. In this study, we found it to be 0.78.

#### The Center for Epidemiological Studies Depression Scale

Radloff (1977) developed the Center for Epidemiological Studies Depression Scale (CES-D). We used the Korean version of the CES-D, translated and revised by Chon and Rhee (1992). It measures individuals’ depressive experiences over the preceding week, using 20 four-point Likert scale items. The higher the final score, the more depressed the individual. Chon and Rhee (1992) reported a Cronbach’s α of 0.89 for the scale while we found it to be 0.92.

#### The Aggression Scale

We used the Aggression Scale originally developed by Buss and Durkee (1957), which was translated and validated by Noh (1983), and revised by Choi (2002). We employed five sub-scales: *physical aggression*, *indirect aggression*, *verbal aggression*, *negativity*, and *excitability*. Participants rated each item on a five-point Likert scale. Higher sum scores indicated stronger aggression. In this study, we found the Cronbach’s α to be 0.71.

### Data Analysis

We conducted data analysis using the IBM SPSS Statistics 20 and AMOS 20. First, we calculated the Cronbach’s αs to measure the reliability of each scale we used. Second, we conducted independent sample *t* tests to examine the differences between the two groups. Third, we derived descriptive statistics including means, standard deviations, skewness, and kurtosis. Fourth, we conducted a Pearson’s correlation analysis to assess the interrelationships between variables. Fifth, we analyzed the validity of the model using a structural equation model to identify the paths of potential variables. We used the DMCP of MPS sub-scales as observation variables and conducted item parceling for other variables to establish observation variables. Sixth, we calculated fit indices of χ^2^, comparative fit index (CFI), Tucker–Lewis index (TLI), and root mean square error of approximation (RMSEA). Seventh, we conducted bootstrapping analysis to verify the significance of the indirect paths between variables. We confirmed the indirect effects at the 95% confidence interval.

## Results

### DMCP of MPS

Table 1 shows the results of the paired *t* tests we conducted to determine the differences based on who assessed mothers’ parenting styles. The differences between the perceptions of mothers and children were not significant (*t* = 1.74, *p* > 0.05). When we conducted paired *t* tests on the sub-variables, we found significant differences in the *love–hostility* (*t* = 2.00, *p* < 0.05) and *rationality–irrationality* (*t* = 3.57, *p* < 0.001) dimensions. This suggests that mothers consider their own parenting styles more affectionate and more reasonable than their children perceive them to be.

**TABLE 1:** Differences between mothers’ and children’s perceptions of mothers’ parenting styles.

Variable	Mother (*N* = 233)		Adolescent (*N* = 233)		DMCP of MPS		Paired- *t*
	*M*	*SD*	*M*	*SD*	*M*	*SD*	
Total parenting style	3.73	0.32	3.68	0.50	0.05	0.42	1.74
1. Love vs. Hostility	4.02	0.41	3.96	0.57	0.06	0.47	2.00*
2. Autonomy vs. Control	3.44	0.48	3.39	0.61	0.05	0.60	1.40
3. Achievement vs. Non-achievement	3.73	0.50	3.75	0.51	−0.02	0.59	−0.47
4. Rationality vs. Irrationality	3.71	0.48	3.56	0.70	0.15	0.63	3.57***

*p < 0.05, ***p < 0.001.

Next, we examined the correlations between the perceptions of mothers and children on the sub-variables. We found that the correlation coefficient of *love–hostility* was 0.57 (*p* < 0.01), while the correlation coefficient of *autonomy–control* was 0.43 (*p* < 0.01). The correlation coefficient of *achievement–non-achievement* was 0.31 (*p* < 0.01), and that of *rationality–irrationality* was 0.48 (*p* < 0.01).

Next, we divided participants into two groups based on mothers’ parenting style evaluation scores and examined whether there were differences between the groups in terms of children’s depression, aggression, and ego-resilience. In group A, mothers’ ratings of their parenting styles were higher than their children’s ratings, and in group B, children’s ratings of their parents’ parenting styles were higher than their mothers’ ratings. We conducted *t* tests to examine whether there were differences in depression, aggression, and ego-resilience (see Table 2). We found that group A had higher depression (*t* = 5.39, *p* < 0.001) and higher aggression (*t* = 4.20, *p* < 0.001) than group B. In addition, group A had lower ego-resilience than group B (*t* = −4.14, *p* < 0.001). In other words, when mothers rated their parenting styles more favorably than their children did, their children were more depressed and aggressive and experienced lower ego-resilience.

**TABLE 2:** Differences in depression, aggression, and ego-resilience between groups A and B.

Variable	Group A (*N* = 118)		Group B (*N* = 115)		*t*
	*M*	*SD*	*M*	*SD*	
Depression	1.88	0.55	1.53	0.43	5.39***
Aggression	3.01	0.43	2.77	0.43	4.20***
Ego-resilience	2.73	0.53	3.02	0.52	−4.14***

Group A: mothers’ ratings > children’s rating, Group B: mothers’ ratings < children’s rating. ***p < 0.001.

Based on these results, we calculated DMCP of MPS by subtracting children’s perceptions from their mother’s perceptions, which was the same approach used in previous studies.

### Correlation Analysis

Table 3 presents the correlation analysis results and the descriptive statistics. Our analysis showed that DMCP of MPS was negatively correlated with ego-resilience (*r* = −0.24, *p* < 0.01), but positively correlated with depression (*r* = 0.38, *p* < 0.01), and aggression (*r* = 0.31, *p* < 0.01). We found that ego-resilience had a significant negative correlation with both dep*re*ssion (*r* = −0.47, *p* < 0.01) and aggression (*r* = −0.28, *p* < 0.01). Meanwhile, depression was positively correlated with aggression (*r* = 0.40, *p* < 0.01). All of the DMCP of MPS on the sub-variables showed significant correlations with depression, aggression, and ego-resilience except one; DMCP-Achievement was not correlated with aggression (*r* = 0.12, *p* > 0.05).

**TABLE 3:** Correlation between observed variables.

Variable	1	2	3	4	5	6	7	8
1. DMCP of MPS	1.00							
2. DMCP-Love	0.81**	1.00						
3. DMCP-Autonomy	0.75**	0.45**	1.00					
4. DMCP-Achievement	0.56**	0.43**	0.17**	1.00				
5. DMCP-Rationality	0.84**	0.59**	0.54**	0.26**	1.00			
6. Depression	0.38**	0.31**	0.36**	0.18**	0.30**	1.00		
7. Aggression	0.31**	0.25**	0.25**	0.12	0.31**	0.40**	1.00	
8. Ego-resilience	−0.24**	−0.22**	−0.18**	−0.16*	−0.17**	−0.47**	−0.28**	1.00
Mean	0.05	0.06	0.05	–0.02	0.15	1.71	2.89	2.87
*SD*	0.42	0.47	0.60	0.59	0.63	0.52	0.45	0.54
Skewness	0.39	0.32	0.06	0.16	0.27	0.90	0.12	–0.15
Kurtosis	0.24	0.14	–0.05	0.90	0.35	0.39	0.79	–0.51

DMCP-Love: Love vs. Hostility, DMCP-Autonomy: Autonomy vs. Control, DMCP-Achievement: Achievement vs. Non-achievement, DMCP-Rationality: Rationality vs. Irrationality. *p < 0.05, **p < 0.01.

Maximum likelihood, a structural equation model-based parameter estimation method, can be applied when the variables fit a normal distribution. Therefore, we examined the skewness and the kurtosis of the variables. If the absolute value of the skewness is greater than 2 or the kurtosis absolute value is greater than 7, a normal distribution cannot be assumed (Klein et al., 2011). We found that the skewness of our data was −0.15 to 0.90 and the kurtosis was −0.51 to 0.90, so the assumption of a normal distribution was established.

### Measurement Model Verification

We examined the suitability of the structural models for DMCP-Love and DMCP-Rationality. The measurement model in DMCP-Love did not have significant suitability. For the DMCP-Rationality measurement model, we conducted confirmatory factor analysis to verify the relationships between the latent and measured variables. The model fit indices were χ^2^ = 68.889 (*p* < 0.001), *df* = 30, TLI = 0.948, CFI = 0.965, and RMSEA = 0.075. Therefore, we judged the fit of the measurement model to be good.

### Structural Equation Model Verification

Next, we verified the structural equation model (SEM). In the SEM, we set DMCP-Rationality as the predictive variable, depression and aggression as the dependent variables, and ego-resilience as the mediating variable. The SEM fit indices were χ^2^ = 68.889 (*p* < 0.001), *df* = 30, TLI = 0.948, CFI = 0.943, and RMSEA = 0.075, which we judged to be good.

Our analysis of the path between potential variables in the SEM showed that DMCP-Rationality negatively affected ego-resilience (β = −0.205, *p* < 0.01) and positively affected aggression (β = 0.213, *p* < 0.01) and depression (β = 0.206, *p* < 0.001). This means that as DMCP-Rationality increases, ego-resilience decreases while depression and aggression increase. Ego-resilience had a significant negative effect on depression (β = −0.503, *p* < 0.001), but a non-significant effect on aggression (β = −0.123, *p* > 0.05). In addition, depression positively affected aggression (β = 0.369, *p* < 0.001).

In the SEM, we found that ego-resilience did not significantly affect aggression (β = −0.123, *p* > 0.05). This result led us to reject the hypothesis we based on previous research and enabled us to make our theoretical research model more concise (Moon, 2009). Therefore, we developed a modified research model by removing the paths between latent variables that were not statistically significant.

We judged the fit of the modified research model to be good based on the following model fit indices: χ^2^ = 70.591 (*p* < 0.001), *df* = 31, TLI = 0.949, CFI = 0.965, and RMSEA = 0.074. Since we found the fits of the two models to be at similar levels, we were able to compare the models by verifying the χ^2^ difference (Δχ^2^) (Yu, 2015). Both models showed good fits, but we selected the modified research model as the final model because it was simpler than the previous model.

**FIGURE 2:**
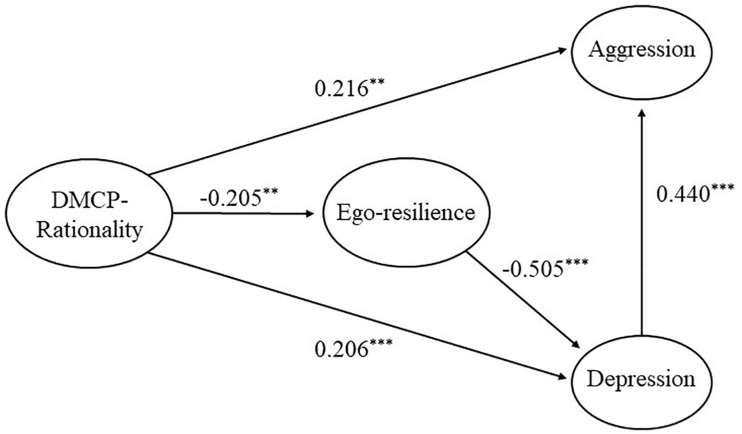
The final model’s path. All estimates are standardized coefficients. **p < 0.01, ***p < 0.001.

Table 4 and Figure 2 present results that confirm the paths between potential variables in the final SEM. Our analysis of the paths between potential variables in the SEM showed that DMCP-Rationality negatively affected ego-resilience (β = −0.205, *p* < 0.01) and positively affected aggression (β = 0.216, *p* < 0.01) and depression (β = 0.206, *p* < 0.001). This means that as DMCP-Rationality increases, ego-resilience decreases while depression and aggression increase. Ego-resilience had a significant negative effect on depression (β = −0.582, *p* < 0.001), and depression positively affected aggression (β = 0.314, *p* < 0.001).

**TABLE 4:** Path coefficients of the final model.

Route		*B*	β	*SE*	*t*
DMCP-Rationality	→ Ego-resilience	–0.151	–0.205	0.054	−2.814**
	→ Aggression	0.131	0.216	0.044	2.964**
	→ Depression	0.175	0.206	0.051	3.425***
Ego-resilience	→ Depression	–0.582	–0.505	0.086	−6.759***
Depression	→ Aggression	0.314	0.440	0.058	5.455***

*B*, unstandardized coefficients; β, standardized coefficients; *SE*, standard error. **p < 0.01, ***p < 0.001.

### Direct Effect, Indirect Effect, and Total Effect Verification of SEM

Using the selected model, we verified the direct, indirect, and total effects. The total effect is the sum of both the direct and indirect effects. We conducted bootstrapping to verify the mediating effects (Shrout and Bolger, 2002). We used 1000 bootstrap samples generated by random sampling from the population (*N* = 233) for parameter estimation. The indirect effect can be considered significant when its 95% confidence interval does not contain 0.

First, the total effect of DMCP-Rationality on depression was significant (β = 0.309, *p* < 0.01). Likewise, the indirect effect of DMCP-Rationality on depression via ego-resilience was significant (β = 0.103, CI = 0.030–0.168, *p* < 0.01). In addition, the direct effect of DMCP-Rationality on depression was significant (β = 0.206, *p* < 0.01). Therefore, our analysis showed that ego-resilience partially mediates the relationship between DMCP-Rationality and depression.

Second, the total effect of DMCP-Rationality on aggression was significant (β = 0.213, *p* < 0.01) as was its indirect effect via ego-resilience and depression (β = 0.028, CI = 0.009–0.058, *p* < 0.01). Likewise, the direct effect of DMCP-Rationality on aggression was significant (β = 0.131, *p* < 0.01). Therefore, DMCP-Rationality directly and indirectly affects aggression through ego-resilience and depression.

Lastly, we examined the single mediating effect of depression and the sequential mediating effect of ego-resilience and depression on the relationship between DMCP-Rationality and aggression. Our analysis showed that depression had a significant mediating effect on the relationship between DMCP-Rationality and aggression (β = 0.055, CI = 0.024–0.103, *p* < 0.01), while ego-resilience and depression had a significant sequential mediating effect on the relationship between DMCP-Rationality and aggression (β = 0.028, CI = 0.009–0.058, *p* < 0.01).

## Discussion

We explored the differences between mothers’ and children’s perceptions of mothers’ parenting styles and the influence of these differences on children’s depression and aggression. Additionally, we examined the mediating effects of ego-resilience and verified the relationships between the variables. In this section, we discuss our results and their implications.

First, we found that mothers rated their parenting styles as more rational and more affectionate than their children did, a finding that aligns with the results obtained in previous studies (Smetana and Asquith, 1994; Chyung, 2002; Kwon, 2009; Yoo, 2018). Studies of elementary school (Kwon, 2009) middle school, and high school (Chyung, 2002; Yoo, 2018) children have consistently found that mothers’ self-evaluations are more favorable and positive than their children’s evaluations of them. One possible explanation for this discrepancy can be found in the generational stakes hypothesis, which explains that parents and children have different interests at different developmental stages and that they see parent–child relationships from different perspectives (Villar et al., 2010). According to the hypothesis, parents try to have positive views of their parenting styles so that their traditions and values can be passed on to subsequent generations, while their children are not positive about their parents’ parenting styles because they are seeking independence and individualization. For instance, a mother may think she has counseled her child as a mentor, but the child may view such counsel as interference (Villar et al., 2010).

Kenny and Albright’s (1987) findings regarding the accuracy of interpersonal perceptions provide another explanation for the discrepancy. They argued that people do not know how they are viewed and evaluated by others especially within well-acquainted groups. In such groups, people tend to perceive themselves more positively than others do, and consequently, the accuracy of their self-evaluations decreases. Regarding implicit personality traits, the study found low correlations between self-evaluations and others’ evaluations. Kenny and Albright’s findings suggest that our results could be attributed to the high familiarity and relatively covert characteristics of rationality and love compared with accomplishment or control. The dimension of rationality in parenting means the raising of children without being emotionally biased by placing importance on rational motives and grounds (Diao, 2011). Therefore, the large difference in rationality between parents and children means that adolescents perceive their parents with fewer reasonable norms and less consistency than their parents perceives themselves to have. Additionally, for adolescents, family bonds become relatively weak as the importance of their peers grows. However, parents still value their relationships with their children and are more likely to perceive these relationships as close and positive than their children. Therefore, our findings underline the importance of parent–child communication, which can decrease differences in perceptions of parenting styles.

Second, we confirmed that DMCP-Rationality significantly affects children’s depression and aggression. While mothers evaluate their own rationality favorably, their children’s evaluations are lower, and the greater the difference, the more depressed and aggressive the children become. These results align with the findings of previous research: inconsistencies in evaluations between mothers and children also negatively affect children’s behavioral problems; in particular, such inconsistencies deteriorate adolescents’ self-directed learning abilities (Kim and Lim, 2012) and increase their problematic behaviors, including withdrawal and hyperactivity (Kim, 2001). The higher the DMCP-Rationality, the more the adolescents’ internalized problems (Yoo, 2018) and negative coping strategies (Cho and Ahn, 2017). Similarly, when there is a large difference in perceptions in emotional communication between parents and children, the children might develop difficulties with emotion control (Ryu, 2016). Therefore, these perceptual differences may represent more than simply differences in evaluations; they may be implicit signs of conflict, tension, and dissatisfaction in mother–child relationships.

Third, we found that ego-resilience plays a mediating role in the relationship between DMCP-Rationality and depression. Previous studies have shown that ego-resilience plays a mediating role in the relationship between mothers’ parenting styles and children’s depression (Park and Yoo, 2011; Park et al., 2014). Researchers have found that mothers’ parenting styles can predict children’s ego-resilience (Wyman et al., 1999; Lee and Shin, 2006; Park and Yoo, 2011; Jeong, 2018). Moreover, when rationality is high, children in junior high experience ego-resilience increases (Ahn et al., 2016). We found that DMCP-Rationality also predicts children’s ego-resilience. Similarly, Cho and Ahn (2017) reported that DMCP-Rationality significantly affects ego-resilience. Ego-resilience refers to the capacity to deal with stressful situations and changes. Parenting styles naturally affect ego-resilience, given that parents typically teach their children coping skills in stressful situations. Furthermore, differences in perceptions of parenting styles also affect ego-resilience. Therefore, DMCP-Rationality can serve as a reliable indicator of coping skills in stressful situations and resulting depression in children.

Fourth, we found that ego-resilience and depression have a double mediating effect in the relationship between DMCP-Rationality and aggression. By revealing these sequential influences, we demonstrated that perceptual differences between mothers and children can ultimately affect children’s aggression. Many researchers have stated that depression precedes aggression (Kim and Baik, 2000; Park and Jung, 2015; Hwang, 2017; Jue and Ha, 2019; Kim, 2019; Nam and Bae, 2019; Lee et al., 2020). We added the influence of DMCP-Rationality and ego-resilience to this sequential relationship. High scores in DMCP-Rationality mean that children feel that their parents lack reasonable standards, motivation, and consistency. Regarding parental rationality, Becker (1964) and Coe (1972) have stated that parents’ inconsistent behavior can lead to children’s maladjustment, conflict, and aggression. Therefore, this finding will have practical implications for educating or counseling youth with adaptation problems.

The significance of this study is as follows. While previous studies have tended to ask children to assess their parents’ parenting styles, we asked both mothers and children to assess parenting styles and examined the differences between these assessments. Based on our findings, we suggest that such perceptual differences could be used as an index to predict children’s psychological health. In addition, while previous studies examined the relationship between perceptual differences and specific factors, we identified a more detailed path between these variables. In other words, when parents are perceived to provide reasonable motives and a solid basis for their children while raising them in a consistent manner, their children experience less depression or aggression, and their ego-resilience increases. Consequently, they become less aggressive. In this way, rational parenting exerts many beneficial influences. Therefore, parents should endeavor to make their parenting rational and react sensitively to their children’s needs so that both parents and children can perceive the parenting as rational. Furthermore, parents will have to make constant efforts to fully communicate with their children while presenting logical reasons and grounds.

This study’s limitations and our suggestions for future research are as follows. First, we used data for Korean mothers and adolescents. Since Korea is a highly competitive society and academic competition is fierce, generalizing the results to people in other countries is difficult. Subsequent studies could increase sample representativeness and generalizability by considering more diverse samples. Second, we focused on the parenting styles of mothers because mothers have been shown to exert stronger psychological effects on their children than fathers (Yoo, 2018), and they reportedly spend more than twice as much time with their children as fathers (Korean Ministry of Health and Welfare, 2018). In this vein, Chyung (2002) found a positive relationship between high perceptual disagreement about the warmth of mothers’ parenting styles and the antisocial behavior of adolescents, but found no such relationship between perceptual disagreement about the warmth of fathers’ parenting styles and the antisocial behavior of adolescents. Likewise, a study of the relationship between middle school students’ perceptions of parenting styles and levels of depression found that fathers’ parenting styles had no significant effect (Ku, 2013). Therefore, we focused on adolescents’ perceptions of their mothers’ parenting styles. Third, while there were differences in DMCP sub-variables, they were small or moderate. Given the accuracy dimension of interpersonal perceptions, the degree of consensus among appraisers depends on many factors including similarity in meaning systems or the familiarity of evaluators (Kenny, 1991). To interpret the meaning of differences in evaluations accurately, future studies would benefit from considering the various factors that affect consensus. Fourth, we investigated the relationship between variables at a specific time and did not observe changes over time. Future studies should more closely examine the interrelationships among variables through longitudinal observation. Finally, we used self-report questionnaires to collect data, which means social desirability bias might have affected the responses. This highlights the limitations of accurate evaluations. Future studies would benefit from utilizing qualitative interviews, which would allow for detailed descriptions of participants’ perceptions.

## Data Availability Statement

The datasets presented in this article are not readily available because they contain information that could compromise research participant privacy/consent. Requests to access the datasets should be directed to hajung366@hanyang.ac.kr.

## Ethics Statement

Ethical review and approval was not required for the study on human participants in accordance with the local legislation and institutional requirements. Written informed consent to participate in this study was provided by the participants’ legal guardian/next of kin.

## Author Contributions

JC and JH conceived the presented idea, developed the theory, and conducted the survey. JH and JJ verified the analytical methods. JC, JH, and JJ analyzed the data. JC and JJ wrote the manuscript in consultation with JH. All authors contributed to the article and approved the submitted version.

## Conflict of Interest

The authors declare that the research was conducted in the absence of any commercial or financial relationships that could be construed as a potential conflict of interest.
